# Synthesis and Characterization of Spirocyclic Mid-Block Containing Triblock Copolymer

**DOI:** 10.3390/polym15071677

**Published:** 2023-03-28

**Authors:** Suraj Aswale, Minji Kim, Dongwoo Kim, Aruna Kumar Mohanty, Heung Bae Jeon, Hong Y. Cho, Hyun-jong Paik

**Affiliations:** 1 Department of Polymer Science and Engineering, Pusan National University, Busan 609-735, Republic of Korea; 2 Department of Chemistry, Kwangwoon University, Seoul 138-727, Republic of Korea

**Keywords:** ATRP, cyclic polymer, liquid chromatographic analysis, block copolymer

## Abstract

Polymers containing cyclic derivatives are a new class of macromolecular topologies with unique properties. Herein, we report the synthesis of a triblock copolymer containing a spirocyclic mid-block. To achieve this, a spirocyclic polystyrene (cPS) mid-block was first synthesized by atom transfer radical polymerization (ATRP) using a tetra-functional initiator, followed by end-group azidation and a copper (I)-catalyzed azide-alkyne cycloaddition reaction. The resulting functional cPS was purified using liquid chromatography techniques. Following the esterification of cPS, a macro-ATRP initiator was obtained and used to synthesize a poly (methyl methacrylate)-*block*-cPS-*block*-poly (methyl methacrylate) (PMMA-*b*-cPS-*b*-PMMA) triblock copolymer. This work provides a synthetic strategy for the preparation of a spirocyclic macroinitiator for the ATRP technique and as well as liquid chromatographic techniques for the purification of (spiro) cyclic polymers.

## 1. Introduction

In recent years, cyclic polymers have attracted attention in polymer chemistry because of their unique topologies. In particular, the absence of chain ends in cyclic polymers results in improved properties, such as a higher glass transition temperature, lower intrinsic viscosity, lower hydrodynamic volume, etc., compared to those of their linear analogs [1,2,3,4,5,6,7,8]. Over the last two decades, various cyclic topologies, including rings, tadpole-like, theta-shaped, grafted, eight-shaped, and catenated polymers, have been reported [9,10,11,12,13]. In addition, (macro)cyclic polymers have been used as novel building blocks for complex polymer architectures, such as cyclic combs and sunflower and jellyfish-shaped topologies, which show enhanced physical properties [10,14]. The wide range of chemical compositions in the cyclic polymers was potentially applied to lithography, emulsion stabilization, self-assembly, dye extraction, drug delivery, antibacterial properties, and topological gel [15,16,17,18,19,20,21].

The facile synthesis of cyclic polymer topologies has been achieved thanks to advances in reversible-deactivation radical polymerizations (RDRP) [22,23] and copper (I)-catalyzed azide-alkyne cycloaddition (CuAAC) reaction [24]. However, after the cyclization reaction, side products are generated via several routes, including radical coupling termination, loss of chain-end functionalities, and intermolecular reactions. Nevertheless, high molecular weight side products have been successfully separated by fractional precipitation and preparative size exclusion chromatography (prep SEC) [25]. However, the low molecular weight side products (i.e., those having a similar molecular weight to the precursor) are needed to be separated with sophisticated techniques. Most low molecular weight side products are generated via a loss in chain-end functionality and have chromatographic resolutions similar to those of the precursors [26]. In this case, prep SEC is limited to the isolation of cyclic products. Recently, advances in liquid chromatography under critical conditions (LCCC) have enabled the separation of polymer mixtures based on their functionalities, regardless of their molecular weights [27,28]. For example, with an increase in the number of polar functionalities of the polystyrene chains, the elution time increases in solvent gradient liquid chromatography [27]. In addition, the LCCC technique has been successfully applied to the purification of cyclic polymers [29,30]. Spirocyclic or cyclic block-connected polymers with different chemical compositions were reported in several recent papers [31,32]. However, spirocyclic polymers containing different functional groups have not yet been evaluated in purification studies. In particular, to utilize spirocyclic polymers as the key role in the RDRP system (i.e., as the atom transfer radical polymerization (ATRP) initiator), elaborate purification techniques are required. Therefore, characterization and purification using advanced liquid chromatography are essential for employing functional spirocyclic polymers in RDRP.

**Scheme 1:**
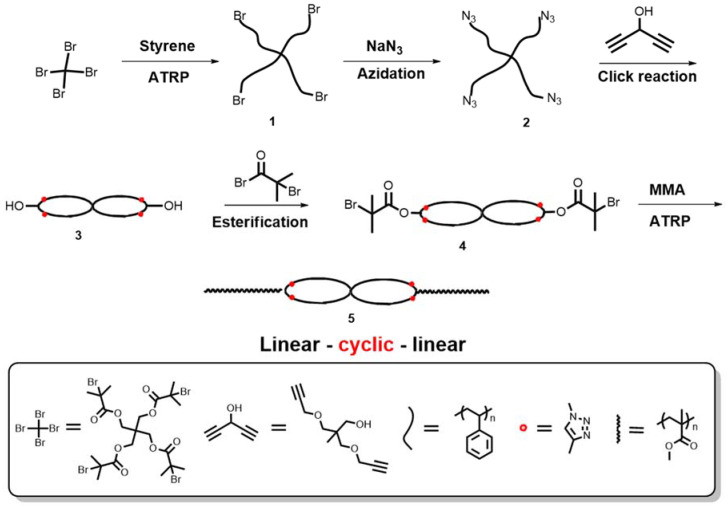
Synthetic route to the spirocyclic midblock contained triblock copolymer.

Herein, we report the synthesis of spirocyclic polystyrene (cPS) as an ATRP initiator to prepare a poly(methyl methacrylate)-*block*-cPS-*block*-poly(methyl methacrylate) (PMMA-*b*-cPS-*b*-PMMA) triblock copolymer (Scheme 1). The cPS in the midblock was synthesized using the ATRP of styrene, azidation of the halogen chain-end, and cyclization via CuAAC. To obtain pure cPS as the spirocyclic macroinitiator, isolation was conducted using prep SEC and medium-pressure liquid chromatography under LCCC. The successful synthesis was confirmed via ^1^H NMR, differential scanning calorimetry (DSC), and matrix-assisted laser desorption ionization time-of-flight (MALDI-TOF) analyses. Finally, the triblock copolymer was prepared via the chain extension of cPS by ATRP with methyl methacrylate (MMA). The synthesis of a well-controlled triblock copolymer was confirmed using ^1^H NMR and Fourier transform infrared (FT-IR) spectroscopy measurements and liquid chromatographic techniques. This synthetic approach provides a route to polymers having tunable physical properties by varying the monomers in the spirocyclic macroinitiator and linear polymers.

## 2. Experimental Section

### 2.1. Materials

Cu^I^Br (Sigma-Aldrich, ≥98%, St. Louis, MO, USA) was purified by stirring with glacial acetic acid for 24 h, followed by filtration. The resulting solid was washed with ethanol and diethyl ether. The solid was dried under vacuum for 48 h. Styrene (Daejung, >99%, Siheung-si, Gyeonggi-do, Republic of Korea) was purified by passing it through a basic alumina column to remove the inhibitor. 2-Bromoisobutyryl bromide (Bibb, Sigma-Aldrich, 98.0%, St. Louis, MO, USA) and Cu^II^Br_2_ (Sigma-Aldrich, 98.0%, St. Louis, MO, USA) were used as received. MMA (Daejung, 99%, Siheung-si, Gyeonggi-do, Republic of Korea) was purified by passing it through a basic alumina column. Magnesium sulfate (MgSO_4_, TCI, Tokyo, Japan), *N*,*N*,*N*′,*N*′′,*N*′′-pentamethyl diethylenetriamine (PMDETA, Sigma-Aldrich, 98%, St. Louis, MO, USA), pentaerythritol (Sigma-Aldrich, 99.0%, St. Louis, MO, USA), triethylamine (TEA, Sigma-Aldrich, 99.5%, St. Louis, MO, USA), sodium azide (NaN_3_, TCI, >99.0%, Tokyo, Japan), tetrahydrofuran (THF, Sigma-Aldrich, anhydrous, ≥99.9%, inhibitor-free, St. Louis, MO, USA), anisole (Daejung, 98.0%, Siheung-si, Gyeonggi-do, Republic of Korea), high-performance liquid chromatography (HPLC)-grade tetrahydrofuran (THF, Fisher, 99.9%, Seoul, Republic of Korea), *n*-hexane (Daejung, 99.5%, Siheung-si, Gyeonggi-do, Republic of Korea), acetonitrile (ACN, Daejung, 99.8%, Siheung-si, Gyeonggi-do, Republic of Korea), dichloromethane (DCM, Samchun, 99.9%, Siheung-si, Gyeonggi-do, Republic of Korea), and other chemicals were purchased from Sigma-Aldrich and used as received.

### 2.2. Characterization

^1^H NMR spectra were obtained on 500-MHz Agilent and 400-MHz JEOL superconducting FT-NMR spectrometers using chloroform-*d* (CDCl_3_) and dichloromethane-*d*_2_ (CD_2_Cl_2_) as solvents. Size exclusion chromatography (SEC) experiments were carried out using an Agilent 1100 pump, refractive index (RI) and UV detectors, a PSS SDV precolumn (5 μm; 50.0 × 8.0 mm), and PSS SDV columns (5 μm; 10^5^, 10^3^, and 10^2^ Å; 300.0 × 8.0 mm) with polystyrene standards for calibration. THF was used as the eluent at 40 °C at a flow rate of 1.0 mL min^−1^. Infrared spectra were recorded on a Nicolet 6700 FT-IR spectrophotometer. For recycling preparative SEC, YMC-GPC T30000 and T2000 columns were used. HPLC-grade THF was used as the mobile phase and delivered using a YMC K-50 HPLC pump at a flow rate of 10 mL min^−1^. For preparative medium-pressure liquid chromatography (MPLC) analysis using a silica column (YMC-Pack SIL, 250 × 20 mm, S-5 µm, 12 nm) at room temperature, the mobile phase was a mixture of THF/*n*-hexane delivered by a binary gradient HPLC pump (YMC K-50 HPLC pump) at a flow rate of 13 mL min^−1^. The gradient elution composition was linearly varied from THF/*n*-hexane = 40/60 (*v*/*v*) for 0–15 min to pure THF for the subsequent 60 min. The MPLC samples were prepared at a concentration of 100 mg mL^−1^, and the injection volume was 5 mL. The HPLC analyses were performed using a reverse-phase column (Luna 5u C18(2) 100 Å, 150 × 4.60 mm, 5 μm) at room temperature, and the mobile phase was a mixture of DCM/ACN delivered by a gradient HPLC pump (Shimadzu LC20 AD) at a flow rate of 0.5 mL min^−1^. The gradient elution composition was linearly increased from DCM/ACN = 40/60 (*v*/*v*) for 0–8 min to pure DCM for the following 60 min. HPLC samples were prepared at a concentration of 5 mg mL^−1^. Chromatograms were recorded using an ultraviolet/visible (UV/vis) absorption spectrophotometer (Younglin UV730D) and a light scattering detector using the characteristic absorption at 658 nm (Wyatt, Minidawn). MALDI–TOF analysis was performed using a Bruker Auto flex speed mass spectrometer. The instrument was operated at an accelerating potential of 20 kV in positive mode. Mass calibration was performed using PS standards. *Trans*-2-(3-(4-tert-butylphenyl)-2-methyl-2-propenylidene) malononitrile (DCTB) was used as the MALDI matrix, and silver trifluoroacetate was used as the cationization agent. The MALDI-TOF samples were prepared with a stock solution of the matrix (15 mg mL^−1^), polymer analyte (5 mg mL^−1^), and cationization agent (2 mg mL^−1^) in THF. The stock solutions were mixed in a 5/1/1 volume ratio (matrix/analyte/cation) and deposited onto a MALDI target plate. DSC analyses were performed using a TA Instruments Q100 device operated at 10 °C min^−1^ heating rate under a nitrogen atmosphere.

### 2.3. Syntheses

#### 2.3.1. Tetra-Functional Initiator (Pentaerythritol Tetrakis (2-Bromoisobutyrate))

Pentaerythritol (3 g, 22.03 mmol) was dissolved in dry THF in a round-bottomed flask. TEA (14.74 mL, 105.77 mmol) was added to the flask at 0 °C and stirred for 1 h. Subsequently, 2-bromoisobutyrate (13.07 mL, 105.77 mmol) in THF (20 mL) was slowly added to the flask over 30 min under a nitrogen atmosphere. The reaction mixture was stirred overnight at room temperature and concentrated using a rotary evaporator. The reaction product was extracted with diethyl ether (300 mL) and washed three times with water (200 mL). After drying with MgSO_4_, the organic solvent was evaporated to isolate the crude product. After recrystallization in hexane, a slightly yellowish product was obtained (yield: 9.8 g, 59%). ^1^H NMR (400 MHz, CDCl_3_) δ (ppm) 4.33 (s, 8H) and 1.94 (s, 24H).

#### 2.3.2. Tetra-Arm Polystyrene (**1**)

A 250 mL Schlenk flask was charged with Cu^I^Br (285 mg, 1.98 mmol), Cu^II^Br_2_ (24 mg, 0.107 mmol), and the tetra-functional initiator (1.53 g, 2.09 mmol). The flask was sealed using a glass stopper, evacuated, and backfilled with nitrogen. Deoxygenated styrene (30 mL, 261 mmol), anisole (15 mL), and PMDETA (0.437 mL, 2.095 mmol) were added to the flask via nitrogen-purged syringes. The flask was kept in a preheated oil bath at 80 °C. After 3 h, the reaction was stopped by diluting with THF and exposure to air, and the catalyst was removed by passing the reaction mixture through a neutral-alumina column. The conversion reached 33%, confirmed using ^1^H NMR spectroscopy. After concentrating the solvent, the solution was precipitated in cold methanol by dropwise addition. The white solid polymer (9.25 g, 88% yield) was isolated by filtration followed by drying in a vacuum oven. The number-average molecular weight (*M_n_,_SEC_*) and polydispersity index (*Đ*) of the product measured using SEC were found to be 4.2 kg mol^−1^ and 1.09, respectively. The molecular weight calculated using ^1^H NMR (*M*_n_,_NMR_) was 4.6 kg mol^−1^.

#### 2.3.3. Azide Functionalized Tetra-Arm Polystyrene (**2**)

Compound **1** (8 g, 1.80 mmol), NaN_3_ (586 mg, 9.01 mmol), and DMF (30 mL) were added to a 100 mL round-bottomed flask and stirred for 24 h at 40 °C. The polymer was extracted with chloroform (300 mL) and washed three times with water (3 × 200 mL). The organic layer was concentrated using a rotary evaporator after drying with MgSO_4_. The polymer solution was precipitated in cold methanol and dried in vacuo, yielding a white solid product (7.56 g, 95%). The *M*_n_,_SEC_ and (*Đ*) were found to be 4.2 kg mol^−1^ and 1.09, respectively.

#### 2.3.4. Coupling Agent (CA) of (2,2-Bis [(2-propyn-1-yloxy) methyl]-1-propanol)

A 250 mL round-bottomed flask was charged with NaOH (6.50 g, 162 mmol) and water (20 mL). Then, a solution of 1,1,1-tris(hydroxymethyl)ethane (5.98 g, 49.8 mmol) in dimethyl sulfoxide (DMSO, 60 mL) was added to the flask at room temperature and stirred for 1 h. The mixture was cooled to 0 °C, and propargyl bromide (12.44 g, 104 mmol) was added to the flask for 30 min, followed by 24 h of stirring at room temperature. The reaction mixture was diluted with water and extracted with ethyl acetate. The organic layer was dried over anhydrous MgSO_4_ and concentrated on a rotary evaporator. The product was purified by column chromatography using *n*-hexane/ethyl acetate (70/30), and the product was obtained as a pale yellowish oil (yield: 6 g, 61%). ^1^H NMR (400 MHz, CDCl_3_) δ (ppm): 4.16 (dd, *J* = 14.0, 2.4 Hz, 4H, OC*H*_2_CC), 3.58 (d, *J* = 4.0 Hz, 2H, CC*H*_2_OH), 3.51 (t, *J* = 8.0 Hz, 4H, CC*H*_2_O), 2.45 (t, *J* = 4.0 Hz, 1H, O*H*), 2.44 (t, *J* = 2.4 Hz, 2H, CC*H*) and 0.90 (s,3*H*). ^13^C-NMR (100 MHz, CDCl_3_) δ (ppm): 79.7, 74.4, 74.0, 68.4, 58.7, 40.4, and 17.4.

#### 2.3.5. cPS (**3**)

A 2 L round-bottomed flask was charged with Cu^I^Br (6.24 g, 43 mmol) and Cu^0^ wire (*l* × *d* = 60 cm × 1 mm). The flask was sealed with a glass stopper, then evacuated, and backfilled with nitrogen (three times). Next, deoxygenated THF (850 mL) was transferred to a flask using a cannula, and then deoxygenated PMDETA (7.53 g, 43 mmol) was injected into the flask using a nitrogen-purged syringe. Compound **2** (2 g, 0.43 mmol) and CA (0.26 g, 1.35 mmol) were dissolved in THF (20 mL) in separate vials and deoxygenized by nitrogen bubbling. Using a syringe pump, the solutions were added slowly to the reaction flask over 10 h at 35 °C, followed by 5 h of stirring, and the reaction was stopped by exposure to the air. The mixture was concentrated using a rotary evaporator and passed through a neutral alumina column to remove the catalyst. Finally, the product was purified using prep SEC and MPLC, yielding 32% (640 mg). The *M*_n_,_SEC_ and (*Đ*) were found to be 3.5 kg mol^−1^ and 1.03, respectively.

#### 2.3.6. Spirocyclic Macroinitiator (**4**)

Compound **3** (220 mg, 0.06 mmol) was dissolved in DCM (10 mL) in a round-bottomed flask. TEA (43 µL, 0.30 mmol) was then added to the flask at 0 °C and stirred for 30 min. A solution of *α*-bromoisobutyryl bromide (39 µL, 0.30 mmol) in DCM (5 mL) was added dropwise to the flask and stirred overnight at room temperature, and the reaction mixture was diluted with DCM and washed three times with water. The organic layer was dried over anhydrous MgSO_4_ and concentrated on a rotary evaporator. A white solid polymer was obtained after precipitation in cold methanol (218 mg, 92% yield). The *M*_n_,_SEC_ and (*Đ*) were found to be 3.8 kg mol^−1^ and 1.02, respectively.

#### 2.3.7. Triblock Copolymer (PMMA-b-cPS-b-PMMA) (**5**)

Compound **4** (50 mg, 0.013 mmol) and Cu^I^Br were added to a 25 mL flask. The flask was sealed with a glass stopper, evacuated, and backfilled with nitrogen gas three times. Then, deoxygenated MMA (1.05 g, 10.48 mmol), anisole (0.5 mL), and PMDETA were added to the flask through nitrogen-purged syringes. The flask was then placed in a preheated oil bath at 80 °C and stirred for 2 h. The reaction was quenched by dilution with THF and air exposure. The copper catalyst was removed by passing the reaction mixture through a neutral alumina column. After concentration, the solution was precipitated in cold methanol, and a white solid polymer (0.51 g) was isolated by filtration followed by drying in vacuo. The conversion of MMA was determined to be 47% by ^1^H NMR spectroscopy, and the *M*_n,SEC_ and *Đ* were found to be 54.8 kg mol^−1^ and 1.17, respectively.

## 3. Results and Discussion

### 3.1. Tetra-Arm Polystyrene

**Figure 1:**
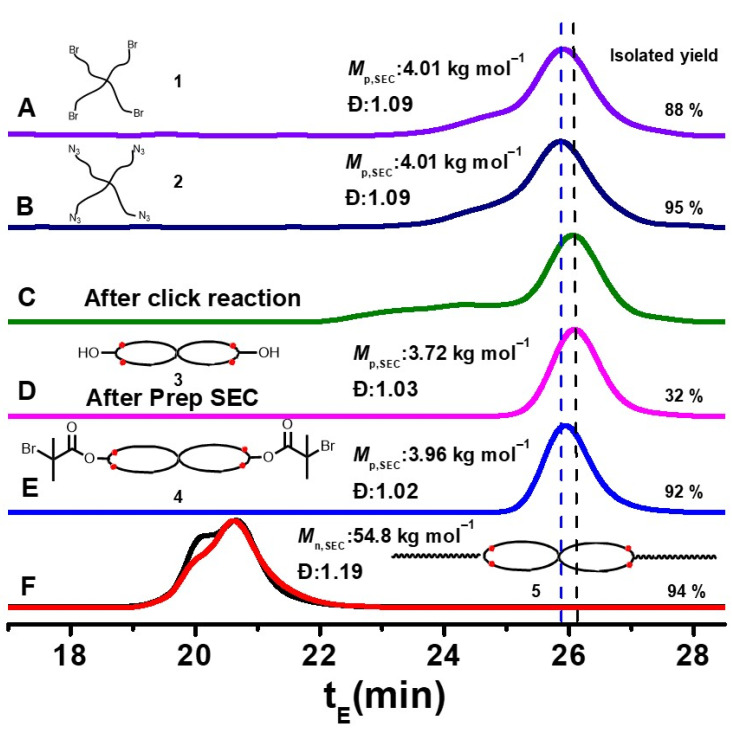
Comparison of the molecular weight with respect to elution time in the SEC measurements (all recorded using RI detector except red line (UV detector)). (**A**) Tetra-arm PS-Br, (**B**) tetra-arm PS-N_3_, (**C**) crude spirocyclic PS, (**D**) after prep SEC spirocyclic PS, (**E**) spirocyclic macroinitiator, and (**F**) PMMA-*b*-cPS-*b*-PMMA triblock copolymer.

Tetra-arm polystyrene (**1**) was synthesized via ATRP using a tetra-functional initiator, pentaerythritol tetrakis(2-bromoisobutyrate). The polymerization was carried out using a 1:125:0.95:0.05:1 molar ratio of initiator, monomer, catalysts, and ligand, respectively (i.e., [initiator]_0_:[styrene]_0_:[Cu^I^Br]_0_:[Cu^II^Br_2_]_0_:[PMDETA]_0_, [styrene]_0_ = 17.45 M in anisole) at 80 °C for 3 h. The *M*_n,SEC_ and *Đ* of **1** were determined using SEC (Figure 1A). 

**Figure 2:**
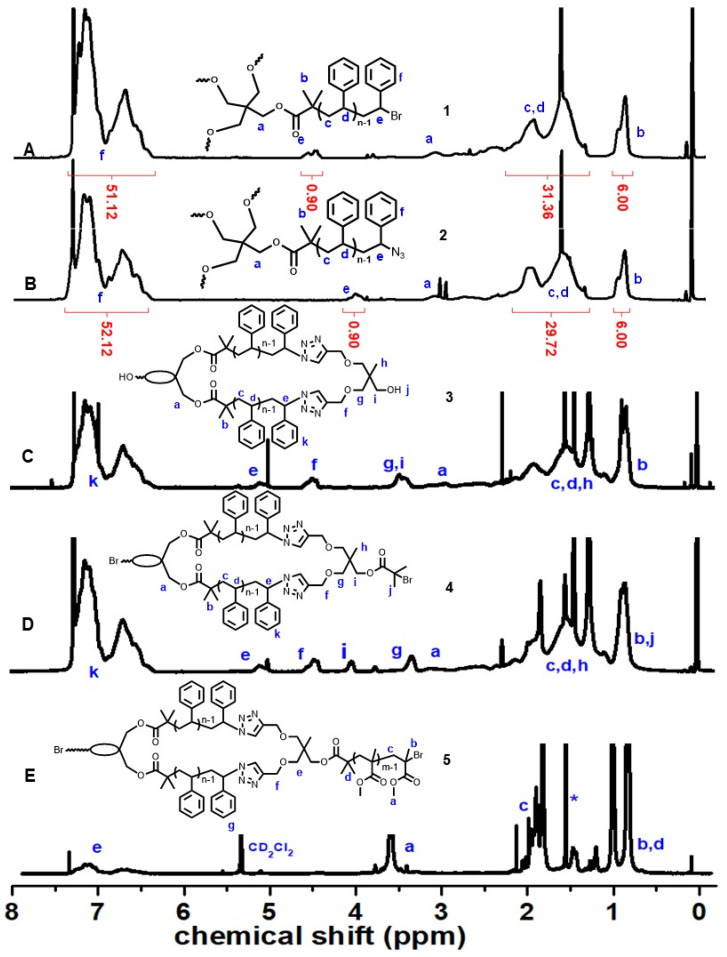
^1^H NMR spectra of (**A**) tetra-arm PS-Br, (**B**) tetra-arm PS-N_3_, (**C**) spirocyclic PS, (**D**) spirocyclic macroinitiator, and (**E**) PMMA-*b*-cPS-*b*-PMMA triblock copolymer (* indicating H_2_O peak).

**Figure 3:**
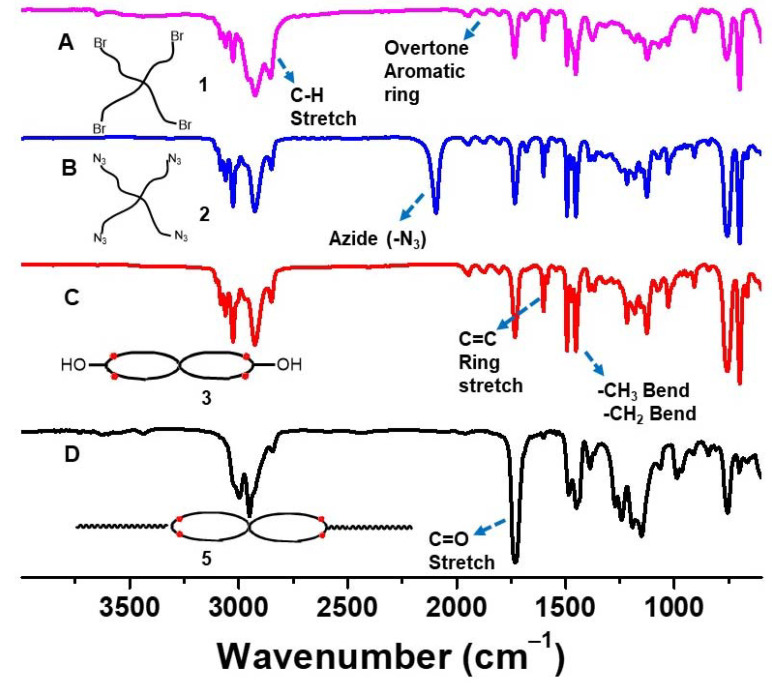
FT-IR spectra of (**A**) tetra-arm PS-Br, (**B**) tetra-arm PS-N_3_, (**C**) spirocyclic PS, and (**D**) PMMA-*b*-cPS-*b*-PMMA triblock copolymer.

Polymer **1** was characterized by ^1^H NMR spectroscopy. The chain-end bromine functionality was calculated by comparing the signals of the methyl (b, -C*H*_3_) and methine (e, -C*H*(C_6_H_5_)Br) protons and was found to be approximately 90% (Figure 2A). The number-average molar weight (*M*_n,NMR_) was calculated by comparing the signals corresponding to the methyl (b, -C*H*_3_) and phenyl (f, -C_6_*H*_5_) protons and was found to be 4.5 kg mol^−1^. Thus, each arm in **1** possessed the number averaged degree of polymerization (DP) of 9. Subsequently, the bromine chain end was converted to an azide via a nucleophilic substitution reaction using NaN_3_. SEC (Figure 1B) and ^1^H NMR (Figure 2B) characterization revealed that the chain end functionality (90%) was unaffected by azidation. Further, in the ^1^H NMR spectrum, the terminal methine (e, -C*H*(C_6_H_5_)-N_3_) proton signal shifted to 3.9 from 4.4 ppm after the substitution reaction (see Figure 2A,B for comparison). In addition, the FT-IR spectra also confirmed the successful azidation based on the appearance of an absorbance peak at 2100–2300 cm^−1^ (Figure 3B).

### 3.2. Spirocyclic PS

Spirocyclic PS with pendent –OH groups were synthesized using a CuAAC click reaction between **2** and CA (i.e., 2,2-bis[(2-propyn-1-yloxy)methyl]-1-propanol). The click reaction was conducted with a mixture of [**2**]_0_:[CA]_0_:[Cu^I^Br]_0_:[PMDETA]_0_ = 1:3.1:100:100 ([**2**]_0_ = 0.5 mM in THF) in the presence of a Cu^0^ wire (*l* × *d* = 60 cm × 1 mm). The reaction was conducted in a diluted system with an excess catalyst to accelerate intramolecular cyclization and minimize side reactions [33]. The formation of **3** was confirmed by SEC with a shift in the peak molecular weight (*M*_p,SEC_) to the lower molecular weight region because of the transformation into a more compact architecture (see Figure 1B,C for comparison) [34]. The *M*_p,SEC_ of **2** and **3** were observed to be 4.08 and 3.72 kg mol^−1^, respectively. The undesired higher molecular weight branched side product in SEC was successfully removed using prep SEC after recycling three cycles (Figure S1). The removal of higher molecular weight branched side products was confirmed by SEC (Figure 1D).

**Figure 4:**
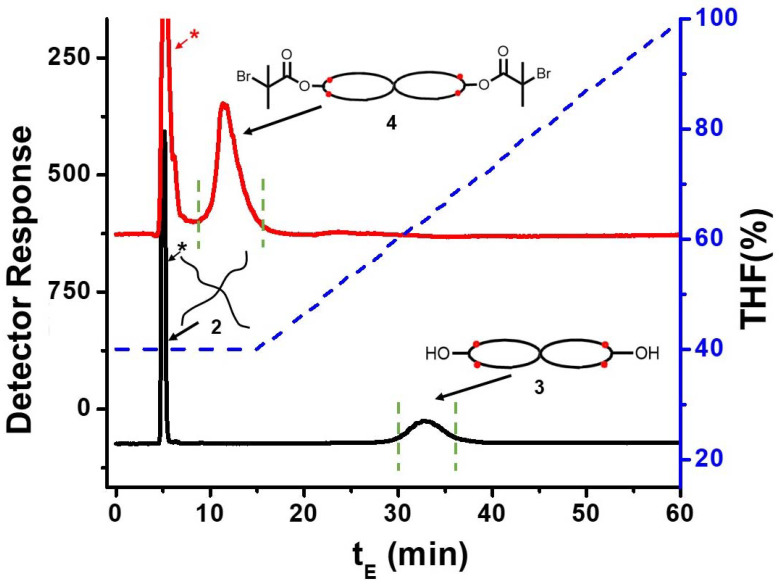
MPLC analysis of spirocyclic polystyrene (black line) and spirocyclic macroinitiator (red line). * indicates solvent peak. Precursor (2, tetra-arm PS-N_3_) is overlapped with solvent (see black line).

**Figure 5:**
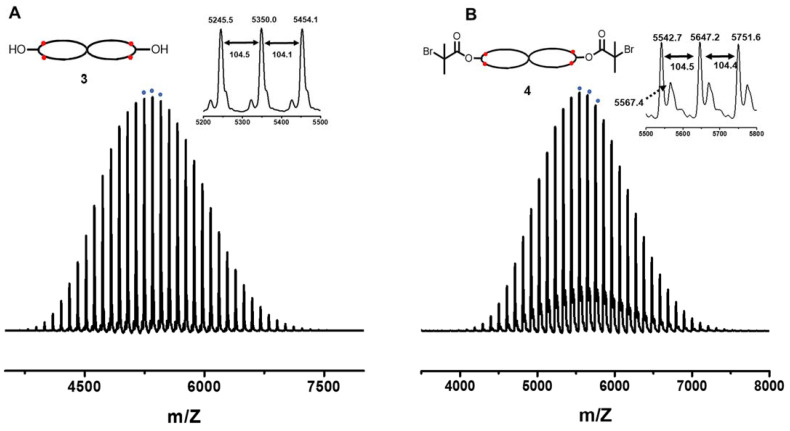
MALDI-TOF mass spectrum recorded using *trans*-2-(3-(4-*tert*-butylphenyl)-2-methyl-2-propenylidene) malononitrile (DCTB) and sodium trifluoroacetate as a matrix and cationization agent, respectively, for (**A**) spirocyclic PS and (**B**) the spirocyclic macroinitiator.

**Figure 6:**
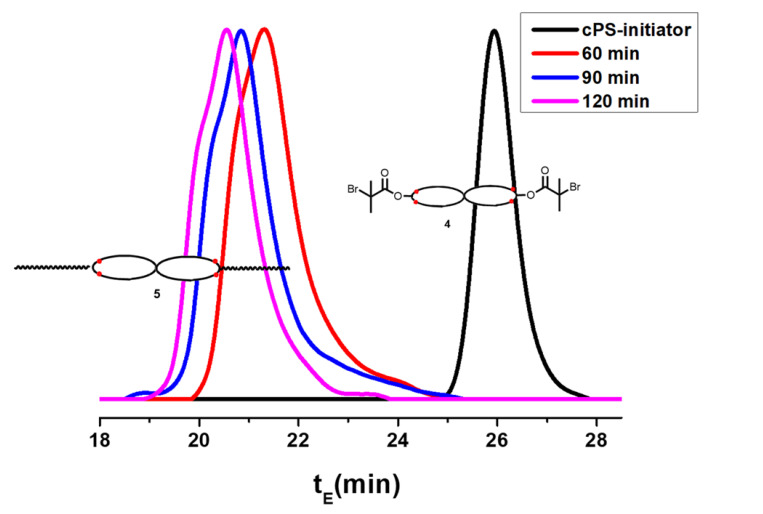
SEC analysis during the chain extension on spirocyclic PS via ATRP of MMA.

However, prep SEC is limited to the separation of mixtures of low molecular weight polymers (e.g., a mixture of cyclic polymers and unreacted precursors) [25] because of their similar retention times (*t*_E_) [26]. This separation can be realized via LCCC, in which polymers are allowed to be separated according to their chemical composition regardless of their molar masses [35]. For instance, hydroxy-functionalized PS has been eluted in a solvent mixture of THF/*n*-hexane (40/60) via the interaction between the polar groups of PS and the stationary phase [28,36,37]. In addition, LCCC has been successfully employed in the MPLC for scale-up separation [38]. Therefore, purification was performed using MPLC with a UV detector (254 nm wavelength) in a mixture of THF/*n*-hexane (40/60) as the mobile phase. Under these conditions, the unreacted precursor was eluted at a similar *t*_E_ as the solvent within 5 min [28], whereas the polystyrene with pendent –OH groups was eluted between 30 and 36 min (black line, Figure 4). The fractionated samples obtained at *t*_E_ values between 30 and 36 min were analyzed by ^1^H NMR, MALDI-TOF MS, and FT-IR spectroscopy. In the ^1^H NMR spectra (Figure 2C), the signal corresponding to the methine proton (e, -C*H*(C_6_H_5_)N_3_) of **2** shifted from 3.98 to 5.08 ppm (see Figure 2B for comparison) and methylene protons (f, -C*H*_2_O-) adjacent the triazole group appeared at 4.48 ppm. The methylene protons (g and i, -C*H*_2_O-) of the CA residue appeared at 3.47 ppm. The molar mass analyzed by MALDI-TOF MS found the *m/z* value for the 41-mer to be 5350.0, which is consistent with the calculated value (*m/z*_cal_ 5350.9, C_371_H_392_O_14_N_12_Ag^+^) (Figure 5A). A repeating mass unit of approximately 104 confirmed the formation of the polystyrene-based product. Further, in the FT-IR spectra, the disappearance of the azide absorbance peak at 2100–2300 cm^−1^ confirmed the complete consumption of the azide during the cyclization reaction (see Figure 3B,C for comparison). Another distinct evidence for the successful transformation into a cyclic polymer is the change in thermal properties. In particular, the glass transition temperature *(T*_g_) of **2** was 76.2 °C, whereas that of spirocyclic PS was 84.6 °C (see Figure 6A,B for comparison). The higher *T*_g_ demonstrates the increased chain rigidity in the absence of the chain ends after cyclization. Collectively, the ^1^H NMR, MALDI-TOF, SEC, FT-IR, and DSC results supported the successful synthesis of spirocyclic PS, **3**.

### 3.3. Spirocyclic Macroinitiator

**Figure 7:**
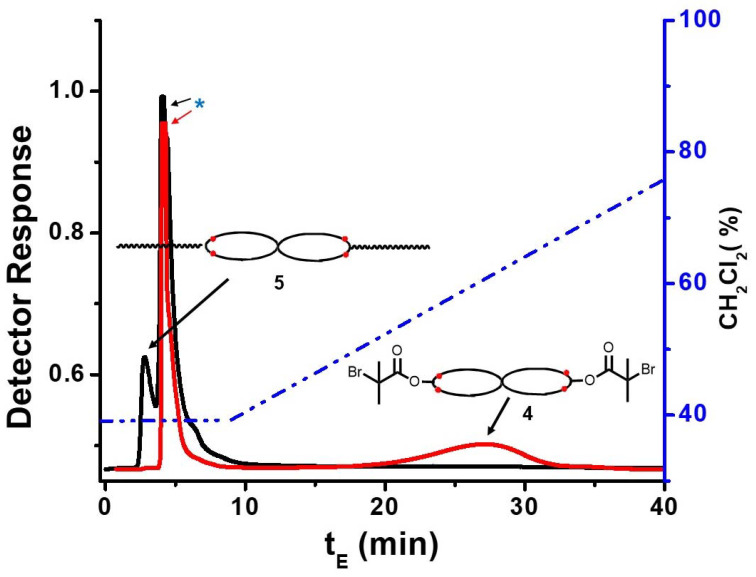
Solvent gradient reverse-phase HPLC fractionation of the spirocyclic macroinitiator and triblock copolymer recorded using a UV detector at 260 nm: spirocyclic macroinitiator (red line), PMMA-*b*-cPS-*b*-PMMA triblock copolymer (black line), and * indicating solvent peak.

**Figure 8:**
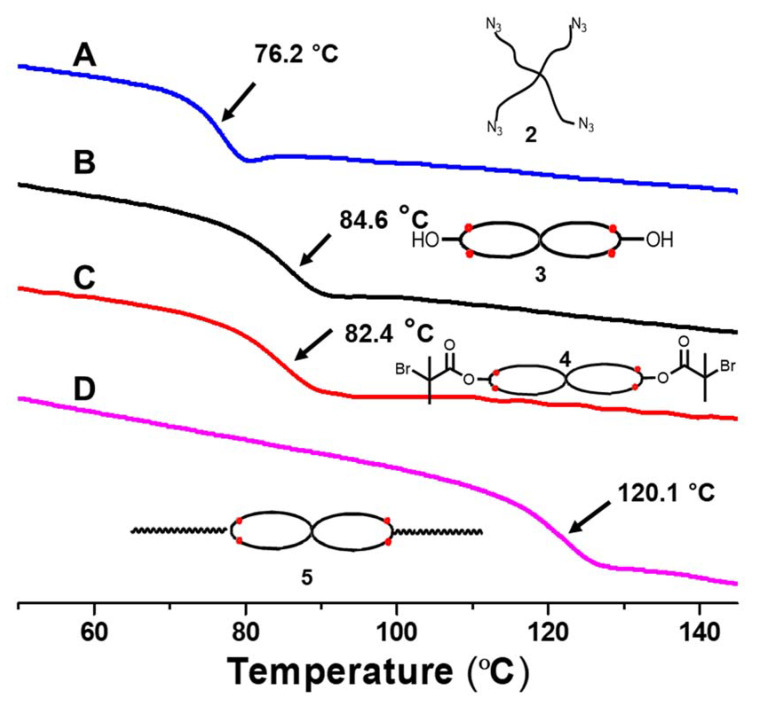
*T*_g_ values of (**A**) tetra-arm PS-N_3_, (**B**) spirocyclic PS, (**C**) spirocyclic macroinitiator, and (**D**) PMMA-*b*-cPS-*b*-PMMA triblock copolymer.

Spirocyclic macroinitiator **4** was prepared by the esterification of **3** with α-bromoisobutyryl bromide in the presence of TEA as a base. The reaction was monitored by thin-layer chromatography (TLC) with an eluent of THF/Hexane (40/60 by volume). The reaction was stopped after 24 h, at which point the complete conversion of the OH functional group had been observed by TLC. After extraction with DCM, the polymer product was characterized using various techniques, including MPLC, ^1^H NMR, SEC, MALDI-TOF, and DSC. During MPLC using THF/hexane (40/60 by volume) as the eluant, the synthesized macroinitiator showed a shorter elution time than compound **3** (*t*_E_ of 9–15 min, red line in Figure 4) (*cf*., *t_E_* of **3** observed between 30 and 36 min) owing to the reduced interaction of the hydroxyl groups with the stationary phase. Also, in reverse phase HPLC (RPLC) the macroinitiator eluted at *t*_E_ = 22−32 min (see red line in Figure 7).The fractionated sample was characterized by ^1^H NMR spectroscopy (Figure 2D), and signals corresponding to the methyl protons of the isobutyryl group were found (j, -C*H*_3_, 1.80–2.00 ppm), whereas a shift in the methylene proton signal (i, -C*H*_2_-, from 3.47 to 4.03 ppm) was observed after esterification (see Figure 2C,D for comparison). In the SEC results, a slight shift in *M*_p,SEC_ from 3.72 to 3.96 kg mol^−1^ was observed (Figure 1D,E for comparison). MALDI-TOF MS analysis (Figure 5B) showed that the *m*/*z* value of the 41-mer was 5647.2, consistent with the corresponding calculated value, *m*/*z*_cal_ 5648.8 (C_379_H_402_O_16_N_12_Br_2_Ag^+^). In addition, a subsidiary peak arising from the loss of HBr was observed at *m*/*z* 5567.4 (*m*/*z*_cal_ 5567.8, C_379_H_401_O_16_N_12_BrAg^+^) during analysis [39]. The *T*_g_ of the spirocyclic macroinitiator was 82.4 °C, which is lower than the values of **3** (see Figure 8B,C for comparison). The *T*_g_ of the resulting compound shows a slight difference due to the chain-end functionality [40]. Thus, all the above characterization results suggested that spirocyclic macroinitiator **4** was successfully synthesized. 

### 3.4. Preparation of the Triblock Copolymer

A triblock copolymer was synthesized by the ATRP of MMA using **4** as the macroinitiator. The reaction was conducted in a mixture of [MMA]_0_:[**4**]_0_:[Cu^I^Br]_0_:[PMDETA]_0_ = 800:1:1:1, ([**4**]_0_ = 0.01 mM in anisole) at 80 °C for 2 h, and the conversion was calculated to be 47% using ^1^H NMR, whereas the conversion-based DP of MMA was 480. The theoretical molecular weight (*M*_n,theory_) of the triblock copolymer was calculated to be 52.0 kg mol^−1^. After passing the crude product through an alumina column, the polymer product was isolated by precipitation in cold methanol and dried in vacuo. The triblock copolymer was analyzed using a solvent gradient (DCM/ACN) RPLC system [41,42] (Figure 7). The *t*_E_ of the triblock copolymer was found between 2.5 and 4.5 min, whereas **4** eluted between 25 and 34 min. In HPLC retention time of a compound depends on its polarity: hydrophilic compounds interact relatively less with the C18 column and elute earlier than hydrophobic compounds [43]. The fractioned sample obtained at a *t*_E_ of 2.5–4.5 min was analyzed using SEC, and it was found to have an *M*_n,SEC_ and *Đ* of 54.8 kg mol^−1^ and 1.19, respectively. The combination of UV (254 nm) and RI detectors in the SEC confirmed the formation of a triblock copolymer (red and black lines in Figure 1F, respectively). Thus, SEC characterization revealed the successful chain extension of **4** with molecular weight evolution during the reaction (Figure 6).

The chemical structure of the triblock copolymer was characterized using ^1^H NMR spectroscopy (Figure 2E). Signals corresponding to the characteristic protons of the methoxy and methyl groups in PMMA and the phenyl group in PS were found at 3.59 (a, -OC*H*_3_), 0.75–1.1 (b, -C*H*_3_), 1.6–1.9 (c, -C*H*_2_-), and 6.2–7.4 ppm (e, -C_6_*H*_5_), respectively. In addition, the PS content in the triblock copolymer, as obtained by ^1^H NMR analysis, was calculated to be 6.4% by comparing the aromatic (6.4–7.2 ppm) and aliphatic (3.5–3.6 ppm) proton signals, and SEC analysis indicated a similar PS content of 7.3%. After copolymerization with MMA, there was an increase in the intensity of the band corresponding to the ester group at 1740 cm^−1^ in the FT-IR spectra (see Figure 3C,D for comparison). Finally, the thermal property of the triblock copolymers was measured using DSC (Figure 8). Owing to the introduction of PMMA into PS, the *T*_g_ of the triblock copolymer (*T*_g_ 120.1 °C) became higher than that of cyclic polymer cPS. The spirocyclic midblock contained triblock copolymer shows slightly higher *T*_g_ than the value of linear triblock copolymer (see Figures S2 and S3 for comparison). The *T*_g_ of triblock copolymer increase may be due to the chain extension of PMMA [44,45]. Collectively, the ^1^H NMR, SEC, FT-IR, HPLC, and DSC analyses indicated that **5** was successfully prepared.

## 4. Conclusions

A triblock copolymer having a unique topology and spirocyclic mid-block was prepared and characterized. The mid-block prepared via a combination of ATRP, azidation, and CuAAC click reaction, was utilized as the initiator for the ATRP of MMA. Crucially, the use of advanced LC techniques (i.e., prep SEC, prep MPLC, and HPLC) allowed the separation of polymers on the chain ends, and polymers having similar molecular weights, such as a 4-arm star (**1** and **2**) and spirocyclic (**3** and **4**) polymers, were successfully isolated using the aforementioned techniques. In addition, the fractionated samples were characterized by SEC, ^1^H NMR, MALDI-TOF, and LCs. Finally, the PMMA-*b*-cPS-PMMA triblock copolymer exhibited a distinguishable *T*_g_ compared to the precursors, as confirmed by DSC, SEC, and HPLC characterization, revealing a successful chain extension. This approach for the synthesis of polymers having novel cyclic topologies using cyclic species as the ATRP macroinitiator will encourage the design of a range of different topological polymers based on a broad choice of monomers.

## Data Availability

Not applicable.

## Supplementary Materials

The following supporting information can be downloaded at: https://www.mdpi.com/article/10.3390/polym15071677/s1, Figure S1. Prep SEC of spirocyclic PS. Figure S2. Molecular weight of linear PS (black line) and linear triblock copolymer (PMMA-b-PS-b-PMMA) (blue and red line for RI and VWD detector, respectively), by SEC. Figure S3. *T_g_* of linear PS (black line) and linear triblock copolymer (PMMA-b-PS-b-PMMA) (red line), spirocyclic macroinitiator (pink line), and triblock copolymer (PMMA-b-cPS-b-PMMA) (blue line).
