# Supplementary material for: Synthesis and Characterization of Spirocyclic Mid-Block Containing Triblock Copolymer
Source: Polymers (Basel). 2023 Mar 28;15(7):1677. doi: 10.3390/polym15071677 (PMC10097252; doi:10.3390/polym15071677)
Supplement: Supplementary file 1 [file polymers-15-01677-s001.zip › polymers-2268373-supplementary.pdf]

Supplementary Material

Synthesis and characterization of spirocyclic mid-block containing triblock copolymer

Suraj Aswale [1], Minji Kim [1], Dongwoo Kim[1], Aruna Kumar Mohanty [1], Heung Bae Jeon [2], Hong Y. Cho [1,*], and Hyun-jong Paik [1,*]

1 Department of Polymer Science and Engineering, Pusan National University, Busan 609-735, South Korea 1; hpaik@pusan.ac.kr

2 Department of Chemistry, Kwangwoon University, Seoul 139-701, South Korea 2; hbj@kw.ac.kr

Correspondence: H.J.P.-hpaik@pusan.ac.kr; H.Y.C- hongycho@pusan.ac.kr

Table of Contents

Figure S1. Prep SEC of spirocyclic PS.

Figure S2. Molecular weight of linear PS and linear triblock copolymer by SEC.

Figure S3. T_g of linear PS and linear triblock copolymer, spirocyclic macroinitiator, and triblock.

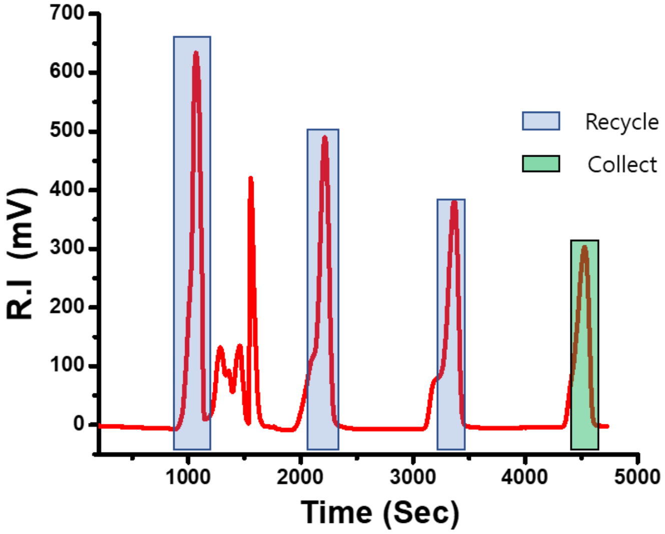

Figure S1. Prep SEC of spirocyclic PS.

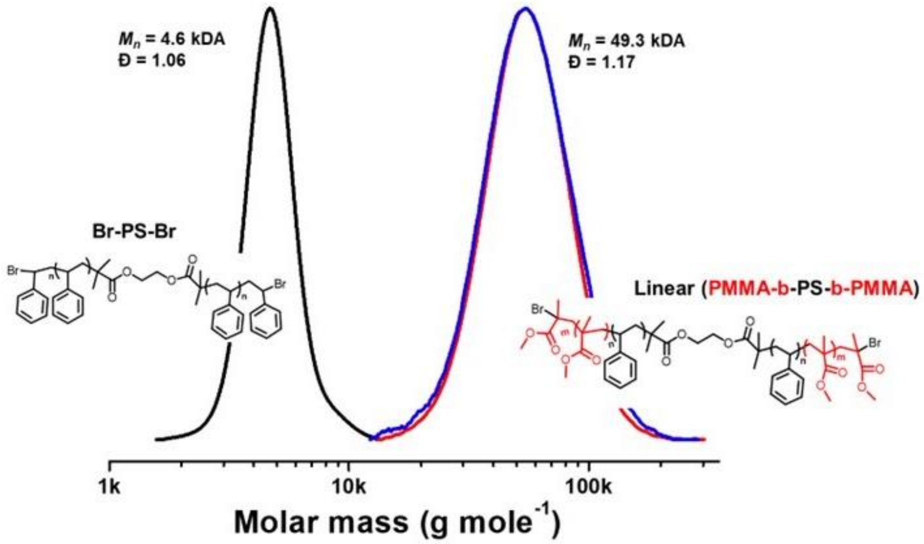

Figure S2. Molecular weight of linear PS (black line) and linear triblock copolymer (PMMA-b-PS-b-PMMA) (blue and red line for RI and VWD detector, respectively) by SEC.

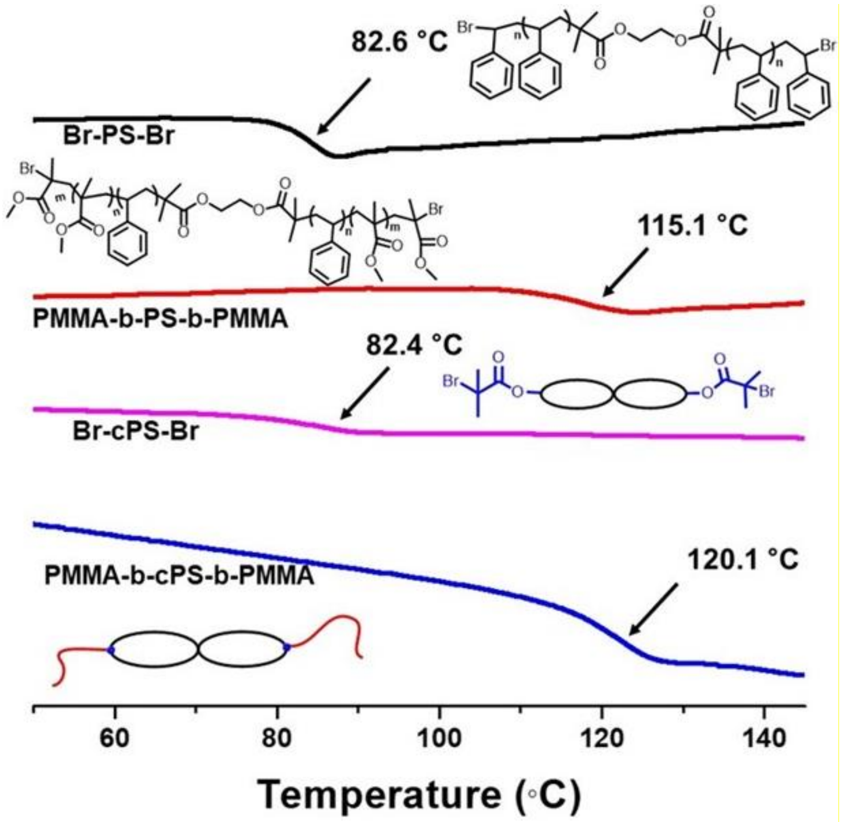

Figure S3. T_g of linear PS (black line) and linear triblock copolymer (PMMA-b-PS-b-PMMA) (red line), spirocyclic macroinitiator (pink line), and triblock copolymer (PMMA-b-cPS-b-PMMA) (blue line).
